# Maternal Risk Factors for Small-for-Gestational-Age Newborns in Mexico: Analysis of a Nationwide Representative Cohort

**DOI:** 10.3389/fpubh.2021.707078

**Published:** 2021-12-23

**Authors:** Suárez-Idueta L, Bedford H, Ohuma EO, Cortina-Borja M

**Affiliations:** ^1^Population, Policy, and Practice Research and Teaching Department, Great Ormond Street Institute of Child Health, University College London, London, United Kingdom; ^2^Maternal, Adolescent, Reproductive and Child Health (MARCH) Centre, Department of Infectious Disease Epidemiology, London School of Hygiene & Tropical Medicine, London, United Kingdom

**Keywords:** low birth weight, small for gestational age, Newborn, maternal educational status, maternal drivers, social determinants of health

## Abstract

**Background:** Small for gestational age (SGA) is a key contributor to premature deaths and long-term complications in life. Improved characterization of maternal risk factors associated with this adverse outcome is needed to inform the development of interventions, track progress, and reduce the disease burden. This study aimed to identify socioeconomic, demographic, and clinical factors associated with SGA in Mexico.

**Methods:** We analyzed administrative data from 1,841,477 singletons collected by the National Information Subsystem of Livebirths during 2017. Small-for-gestational-age was defined as being <10^th^ centiles according to the INTERGROWTH-21^st^ standards. The comparison group was defined as being in ≥10^th^ centiles. We fitted logistic regression models to determine odds ratios for the maternal factors associated with SGA.

**Results:** Among the 1,841,477 singletons, 51% were male, 6.7% were SGA, 6.1% were term-SGA, and 0.5% were preterm-SGA. Maternal education presented a protective gradient of being SGA among mothers who achieved 1 to 6 years of education (adjusted odds ratio (aOR)0.95; 95% CI:0.91,0.99), 7 to 9 years (aOR 0.86; 95% CI:0.83,0.89), 10 to 12 years (aOR 0.75; 95% CI: 0.72, 0.79) and > 12 years (aOR 0.63; 95% CI:0.6,0.66) compared with those without education. SGA was particularly likely to occur among primiparous (aOR 1.42; 95% CI: 1.39, 1.43), mothers living in very high deprivation localities (aOR 1.39; 95% CI: 1.36, 1.43), young (aOR 1.04; 95% CI: 1.02, 1.06), advanced age (aOR 1.14; 95% CI 1.09, 1.19), and mothers living in areas above 2,000 m (aOR 1.69; 95% CI: 1.65, 1.73). Antenatal care was associated with a reduced risk of SGA by 30% (aOR 0.7; 95% CI:0.67,0.73), 23% (OR 0.77; 95% CI:0.74,0.8), and 21% (OR 0.79; 95% CI:0.75,0.83), compared with those mothers who never received antenatal care, when women visited the clinic at the first, second and third trimester, respectively.

**Conclusion:** Almost 7% of live births were found to be SGA. Parity, maternal age, education, place of residence, and social deprivation were significantly associated with this outcome. Antenatal care was protective. These findings imply that interventions focusing on early and adequate contact with health care facilities, reproductive health counseling, and maternal education should reduce SGA in Mexico.

## Introduction

Birth weight is a crucial indicator for the identification and classification of adverse health outcomes at birth (1). The term small for gestational age (SGA) refers to those live births whose weight lies below the 10^th^ percentile compared with infants of the same gestational age and sex. In 2019, SGA, prematurity (<37 weeks of gestation), and low birth weight (<2,500 g) were major contributors to health loss, concentrating 7.3% of all Disability Adjusted Life Years, worldwide (2). In Latin America, SGA has been linked to an increased risk of neonatal mortality (relative risk [RR] 2.62, 95% CI 1.53, 4.49) compared with infants whose birth weight was appropriate for their gestational age (3).

One major challenge to identifying high-risk neonates is the lack of reliable data on birthweight and gestational age. In 2019, UNICEF (4) estimated a higher proportion of live births without recorded weight in registries in low (52.6%) and middle-income countries (37.9%) compared to high-income countries (2.6%). For instance, in Latin America, 7.5% of infants were neither weighed at birth nor had their birth weight recorded in national statistics systems. Furthermore, low, and middle-income countries face significant challenges to accurately determine gestational age and to conduct further assessments regarding the relationship between weight and growth, such as identifying infants' SGA. Thus, the measurement of SGA as a public health indicator is rather limited in this region (5).

Advances in technology have facilitated the availability and study of large datasets. In Mexico, the National Information Subsystem of Livebirths (Subsistema de Información Sobre Nacimientos, SINAC) collects data on births that occurred in health care settings and within the community. This individual-level dataset offers the advantage of including information of newborn babies (e.g., sex, gestational age), mothers (e.g., maternal age, parity, place of residence, marital status), pregnancy follow-up, and delivery (6). Even though the country has achieved substantial improvements in provision for pregnant women of free antenatal and delivery care for all (e.g., health promotion courses, supplementation with iron, folic acid, immunizations, detection of hypertension, diabetes), coverage of this package differs substantially within the country. A study identifying gaps in the continuum of care during pregnancy and delivery in Mexico (7) stated how a more granular description of vulnerable newborns and their mothers should inform public health policy. For instance, it was shown that women living in metropolitan areas have an increase coverage of care (0.87; 95% CI:0.86, 0.89), compared to those in remote areas (0.72; 95% CI:0.7, 0.74) or who self-identified as indigenous (0.75; 95% CI:0.74, 0.77).

While studies have described SGA infants born in the past 20 years in Mexico (8–10), there is no research regarding the use of INTERGROWTH-21^st^ (11) standards in this country. Further description of SGA infants at the national level and their relationship with risk factors may provide evidence for comparing SGA prevalence with other countries and designing context-specific measures to reduce SGA thus contributing toward reducing neonatal deaths. Accordingly, we investigate the association between socioeconomic, demographic, and maternal factors and SGA outcome among a population-representative cohort in Mexico.

## Materials and Methods

### Data Management

Data were obtained from the National Information Subsystem of Livebirths (SINAC for its acronym in Spanish), including all live births that occurred and were registered in Mexico between the 1^st^ of January and the 31^st^ of December in 2017 (6). Raw data with information on live births were extracted as an Office Access file from the SINAC's Hub administered by the Ministry of Health and then processed. Supplementary Table 1 describes the dataset according to the Dublin Core Metadata Initiative's guidelines, which use standardized vocabulary and concepts to encourage the best practices of interoperability, worldwide (12).

Data on the Social Deprivation Index (SDI) for 2015 were extracted from an official open-access website (13) This multidimensional index is calculated every five years by the National Council of Evaluation of Public Policies and summarizes six indicators associated with social deprivation, namely, the number of people without education, people without access to health care, households with essential services, dirty floor, piped water, drainage, electricity, and access to food. We linked information on social deprivation with the state of the mother's residence (14).

**Figure 1:**
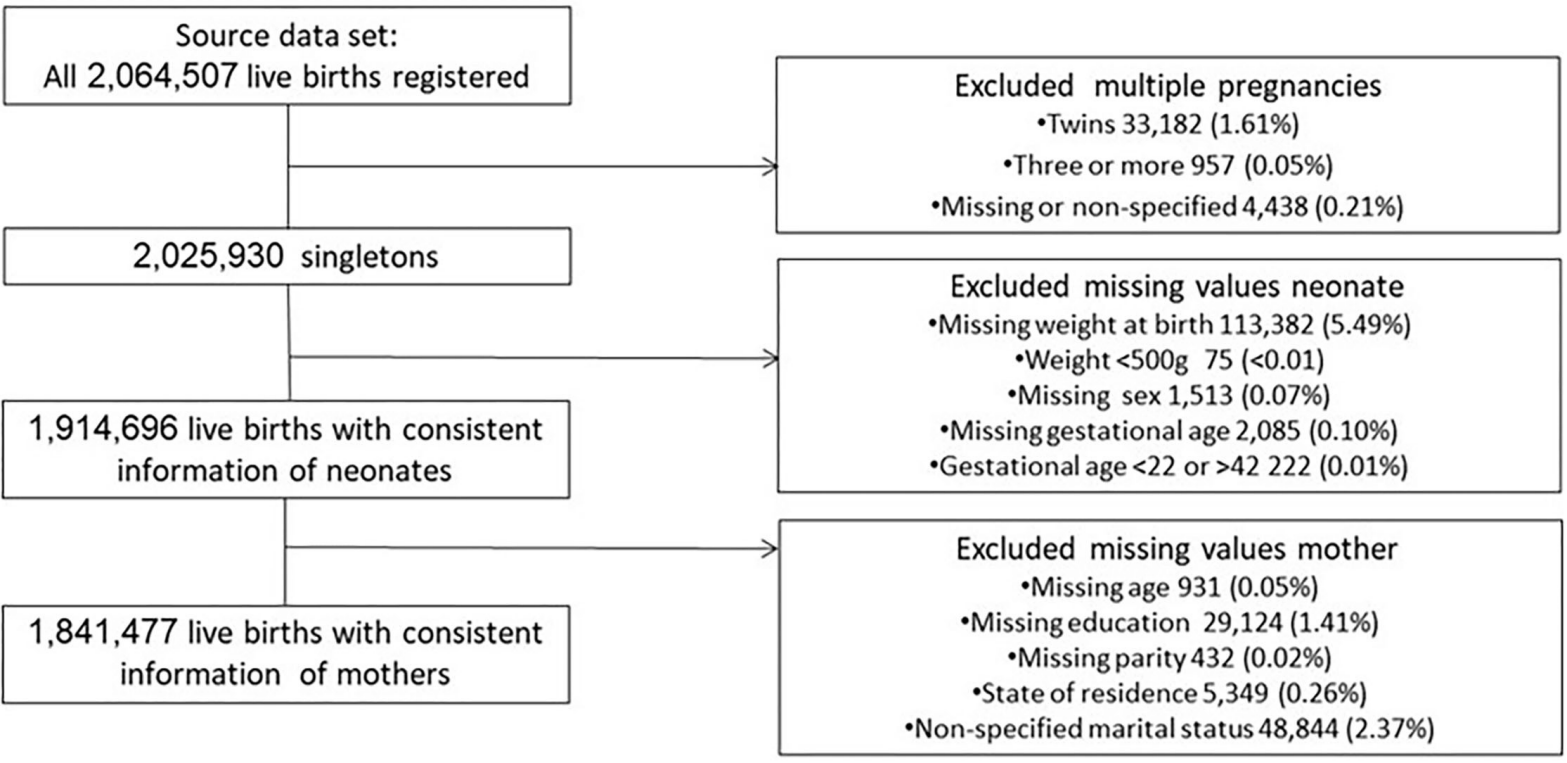
Data flowchart.

Incomplete and outlying values were identified, analyzed, and eliminated from the dataset (Figure 1).

### Data Analysis and Definitions

The Ministry of Health defines livebirth as “the complete expulsion or extraction from a woman of a fetus who presents any signs of life such as heartbeats, pulsations of the umbilical cord, effective and voluntary movements contractions, irrespectively of the gestational length of the pregnancy, whether or not the cord is cut or whether or not the placenta is expulsed” (15). We defined small-for-gestational-age as <10^th^ centile birthweight by sex and completed weeks using the INTERGROWTH-21^st^ international newborn size at birth by sex standards (11). Preterm was defined as <37 gestational weeks, the term was ≥37 weeks and post-term was ≥42 weeks.

We defined coverage as the number of live births reported to SINAC, according to the mother's state of residence, divided by the number of live births estimated by the National Population Council (16), for the same state in 2017.

Maternal baseline characteristics were also analyzed. Young maternal age was defined as <19 years old whereas advanced maternal age was defined as ≥35 years old. Social deprivation was defined according to the National Council of Evaluation of Public Policies as a threshold value ≥1. This index value was also categorized into five levels of social deprivation: Very low, low, medium, high, and very high (14). We defined timely antenatal care (ANC) when women had the first contact with health care services during the first trimester and adequate ANC when women received at least five prenatal visits, according to recommendations of the Ministry of Health (7).

We also reported missing values across different states, describing proportions, and determinants for missingness (Supplementary Table 2).

Our model aimed to summarize the effect of maternal education and relevant determinants on the risk of SGA. We fitted logistic regression models to estimate the probability of being SGA (<10^th^ centiles) according to INTERGROWTH-21^st^ standards. The comparison group was defined as non-SGA (≥10^th^ centiles). Explanatory variables were selected by significance criteria, as suggested by Heinze et al. (17). Model selection was based on Akaike's Information Criterion (AIC) which allowed us to identify the best fit from a group of plausible non-nested models (18). Smaller AIC values indicated improved penalized goodness of fit (19). Also, we used a backward elimination algorithm based on AIC. Our model was finally adjusted by educational attainment, maternal age, marital status, parity, social deprivation, altitude, and antenatal care. Given that INTERGOWTH-21st identifies vulnerable newborns according to sex and gestational age, these two variables were not considered for adjustment. To better understand the effect of prenatal visits on SGA pregnancies, we explored the variable antenatal care as the trimester of first contact with health care services, and we also explored a sensitivity analysis with the number of visits.

All statistical analyses were performed using R programming language and environment for statistical examination and Graphics version 4 (20).

### Research Ethics Approval

No ethical approval was required as the data are anonymized and publicly available (21).

## Results

The primary dataset included 2,064,507 neonates born and registered in Mexico in 2017. Only 2,025,930 (98.1%) records from singletons were included in the principal analysis. We found that weight at birth was the indicator with the highest proportion of missing values (113,382, 5.4%), followed by marital status (48,844, 2.3%), schooling (29,124, 1.4%), state of residence (5,349, 0.26%), gestational age (2,085,0.1%), child sex (1,513,0.07%), maternal age (931,0.05%), and parity (432,0.02%) (Supplementary Table 2). Finally, we analyzed data from 1,841,477 (89.1%) live births with complete information regarding neonates and their mothers. The steps we took to derive the dataset are illustrated in Figure 1.

### Coverage of Live Births Registered in Mexico

**Figure 2:**
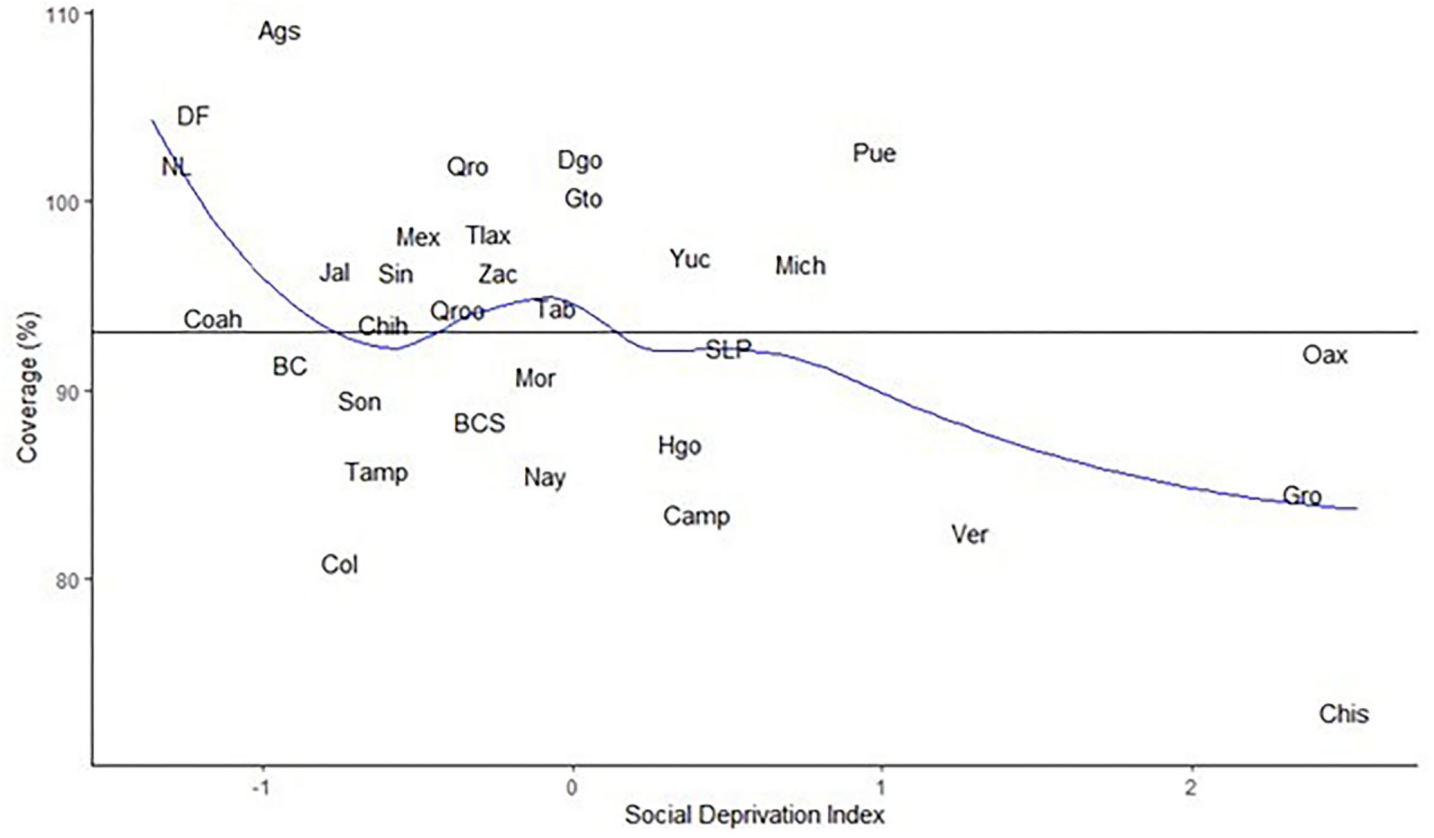
Coverage of notification vs. social deprivation index by state. Mexico, 2017.

At the national level, the National Information Subsystem of Livebirths included 93.3% of the total live births estimated in 2017. Figure 2 summarizes the coverage of live births by state and level of social deprivation. The state of Aguascalientes (Ags), in the center of the country, which had a low deprivation value and reported 8% more live births compared with projections, and Chiapas (Chis), in the southeast, which is the most impoverished state and only included 71% of live births projected by CONAPO appear as possible outliers. The loess smoother in Figure 2 shows how the coverage decreases fast for the least deprived states (SDI < −1) whilst there is a weaker dependence among states with SDI > −1. The black line represents the mean national coverage.

### Characteristics of the Sample

The baseline features of the sample are presented in Supplementary Table 2. Approximately 13% of mothers were young (<19 years old), and 10% were aged ≥35 years. Almost 2% of women did not study with the rest having at least one year of school enrolment. Overall, almost 40% of pregnant women referred to their first pregnancy and 10% were not married nor living with a partner. Nearly a third of mothers lived in areas with high (17.2%) and very high (16.5%) social deprivation. Results by maternal age categories are depicted in Supplementary Table 3. More than 50% of young women referred from 7 to 9 years of education (54.4%), whereas around one-third of women with advanced age referred more than 12 years of schooling. Women in their first pregnancy were more common among young mothers (82.5%), followed by those aged 19 to 34 years (31.1%), between 35 and 39 years (12.2%), and ≥40 years (11.5%). The distribution of baseline characteristics was similar among male and female pregnancies. In 2017, 98% of women identified in the National Registry had access to health care facilities. Of them, 76% visited the clinic during the first trimester and around 84% had five or more prenatal appointments (Supplementary Table 4).

Almost half of the live births were female (49.03%) and the mean weight at birth was 3,148 g (*SD*: 455), with a range of 501 to 6,300 g. Girls had a lower birth weight (3,120 g) than boys (3,177 g). Birth weight also varied by parity and maternal age. Primiparous women had lighter live births compared with mothers who had to at least two pregnancies, especially women aged ≥35 years (3,058 g, *SD* 502) and young women (3,089 g, *SD* 434).

### Small for Gestational Age Infants

We found that 122,607 (6.7%) live births were small for their gestational age according to INTERGROWTH-21^st^ standards of whom 78,920 (64.4%) were men and 43,687 (35.6%) were female. The State of Mexico (9.4%), Yucatan (9.4%), and Guerrero (8.8%) presented the highest prevalence of SGA live births whereas Sonora (3.1%), Sinaloa (3.3%), and Baja California (3.9%), in the North-West of the country, reported the lowest. We also found 9,600 (0.5%) preterm-SGA and 113,007 (6.1%) term-SGA live births (Supplementary Table 5). SGA livebirths were more common in pregnancies between 37 and 41 weeks (88.6%), followed by those preterms between 32 and 36 weeks (7.1%), and post-term pregnancies (3.6%) (Supplementary Table 6).

Table 1 shows the effects of risk factors among SGA infants. The odds of being SGA (vs. non-SGA) was 5% less likely (aOR:0.95; 95% CI:0.91, 0.99) among mothers with elementary education (1–6 years of schooling) compared with women with no education. Maternal education provided a protective gradient of being SGA among mothers who studied 7–9 years (aOR:0.86; 95% CI:0.83, 0.89) 10 to 12 years (aOR:0.75; 95% CI:0.72, 0.79) and more than 12 years (aOR:0.63; 95% CI:0.6, 0.66) compared with those without education.

**Table 1:** Maternal risk factors for SGA infants in Mexico, 2017.

**Variables**	**Unadjusted**		**Adjusted**			
			**Using ANC by trimester**		**Using number of ANC visits**	
	**OR**	**95%CI**	**aOR**	**95%CI**	**aOR**	**95%CI**
**Education, years**
No studies	Reference					
1 to 6	0.91	(0.87, 0.95)	0.95	(0.91, 0.99)	0.97	(0.92, 1.01)
7 to 9	0.82	(0.79, 0.86)	0.86	(0.83, 0.89)	0.88	(0.85, 0.92)
10 to 12	0.76	(0.73, 0.79)	0.75	(0.72, 0.79)	0.78	(0.74, 0.81)
>12	0.61	(0.59, 0.64)	0.63	(0.60, 0.66)	0.65	(0.62, 0.67)
**Age, years**
<19	1.34	(1.32, 1.36)	1.04	(1.02, 1.06)	1.04	(1.02, 1.05)
19 to 34	Reference					
35 to 39	0.91	(0.88, 0.92)	0.98	(0.96,1.00)	0.99	(0.97, 1.02)
≥40	1.09	(1.03, 1.13)	1.14	(1.09, 1.19)	1.15	(1.10, 1.20)
**Marital status**
Single	1.12	(1.1, 1.14)	1.03	(1.01, 1.04)	1.02	(1.00, 1.04)
Married	Reference					
**Parity**		
First pregnancy	1.39	(1.37, 1.41)	1.42	(1.39, 1.43)	1.42	(1.40, 1.44)
Second or more pregnancy	Reference					
**Social deprivation index**
Very low	Reference					
Low	1.03	(1.01, 1.05)	1.03	(1.01, 1.05)	1.03	(1.01, 1.05)
Medium	0.94	(0.91, 0.96)	1.06	(1.04, 1.09)	1.06	(1.04, 1.09)
High	1.2	(1.18, 1.23)	1.1	(1.08, 1.13)	1.11	(1.08, 1.13)
Very high	1.18	(1.15, 1.21)	1.39	(1.36, 1.43)	1.39	1.35, 1.42)
**Altitude**
Low (<80m)	Reference					
Mid (80-1,999m)	1.11	(1.08, 1.13)	1	(0.98, 1.02)	1.01	(0.98, 1.03)
High (≥2,000m)	1.7	(1.66, 1.73)	1.69	(1.65, 1.73)	1.70	(1.66, 1.73)
**Visits to health care facilities, trimester**
No visits	Reference					
First	0.64	(0.62, 0.67)	0.7	(0.67, 0.73)		
Second	0.76	(0.73, 0.79)	0.77	(0.74, 0.80)		
Third	0.78	(0.75, 0.82)	0.79	(0.75, 0.83)		
**ANC, number of visits**						
No visits	Reference					
1	0.88	(0.83,0.94)			0.85	(0.80, 0.91)
2	0.91	(0.86, 0.96)			0.93	(0.88, 0.98)
3	0.87	(0.83, 0.91)			0.88	(0.84, 0.92)
4	0.83	(0.80, 0.87)			0.84	(0.81, 0.88)
≥5	0.65	(0.62, 0.67)			0.69	(0.67, 0.72)

*ANC, antenatal care*.

Mothers aged under 19 years and above 39 years had a 4% (aOR: 1.04; 95% CI: 1.02, 1.06) and 14% (aOR: 1.14; 95% CI: 1.09, 1.19) more chance of having SGA babies compared with those aged between 19 and 34 years old. Single mothers had a 3% more chance of SGA (aOR: 1.03; 95% CI: 1.01, 1.04) than married. Primiparous women had a 42% more chance of SGA (aOR: 1.42; 95% CI: 1.39, 1.43) compared with those with two or more pregnancies. Also, socially disadvantaged mothers had a higher risk of having SGA babies, for example, those living in low (aOR: 1.03; 95% CI: 1.01, 1.05), medium (aOR: 1.06; 95% CI: 1.04, 1.09), high (aOR: 1.10; 95% CI: 1.08, 1.13), and very high deprivation (aOR: 1.39; 95% CI: 1.36, 1.43), compared with those living in areas with very low deprivation. Living in high altitude areas (>2,000 m) increased the odds of SGA by 69% (aOR: 1.69; 95% CI: 1.65, 1.73) compared to living at the lowest altitudes (<80 m).

On the other hand, timely antenatal care was protective. Mothers who had their first contact with the ante-natal care clinic during their first trimester had a 30% (aOR:0.7; 95% CI:0.67, 0.73) decreased risk of having SGA newborns, followed by those who visited the clinic during the second, 23% (OR:0.77; 95% CI:0.74, 0.8) and third trimester 21% (OR:0.79; 95% CI:0.75, 0.83), compared with those mothers who never receive antenatal care. Sensitivity analysis showed that the number of visits also showed a reduced risk of SGA. Mothers who had at least five prenatal visits had a 31% less chance of having SGA livebirths (aOR:0.69; 95% CI:0.67, 0.72) compared with those who missed antenatal care.

## Discussion

### Key Findings

We have provided a nationwide description of small-for-gestational-age infants and their determinants in Mexico. In multivariable analysis, we found that lower maternal education, being age extremes, unmarried mothers, living in districts with higher social deprivation and altitude, and with poor antenatal care were statistically associated with SGA in Mexico. Also, we showed that not only the early contact with health services during the first trimester but the adequate number of prenatal visits (at least five) were protective.

### Strengths and Limitations

This is the first study that uses INTERGROWTH-21^st^ standards to identify SGA infants in Mexico. This global reference results from a multicenter, multiethnic, and population-based project conducted in low and middle-income countries (11), then it offers the possibility to standardize newborns' growth, rather than describe how live births have grown at one specific time and location, for this reason, this tool facilitates comparisons across different settings.

The National Information Subsystem of Livebirths offered clear advantages for this investigation such as the possibility to include a higher number of live births than other hospital-based systems because this registry usually receives information from health facilities and infants born within the community (22). We found that more than 90% of live births were represented in the studied dataset when we contrasted with national estimations derived from the number of people enumerated by regular census and harmonized by trends on fertility, mortality, and migration (16).

Despite such high coverage of national live births, the quality of data on birth weight is still a challenge for two reasons. Firstly, 5.4% of live births had no record of birth weight and this might lead to biased, and overestimated SGA rates (23). Secondly, our results may underrepresent vulnerable groups who tend to deliver at home or do not have contact with health care facilities early in life (24). Another key limitation of this administrative dataset is the lack of data on clinical features that have been associated with SGA in other studies, such as maternal BMI, infectious diseases, chronic diseases, smoking, stress, and intrafamily violence (25, 26).

### Interpretation

Our results show differences in birth weight by parity, and maternal age suggesting that the place in the family had a more considerable influence on birth weight than maternal age, as Karn *et al* described from the statistical description of a cohort of 13,730 live births in England (27). Primiparous mothers aged 35 or more had the lightest live births, a possible mechanism is the presence of sclerotic lesions in the uterus and placental under perfusion (28, 29).

We found that 6.7% of live births were SGA. This proportion is much lower than the 13% reported in 2014 by Ota et al. (8) for Mexico. There are four possible reasons to explain this difference. Firstly, our definition of SGA is based on the INTERGROWTH-21^st^ standards which include a large cohort from a multicenter study conducted in eight geographical areas (11) whereas Ota et al. generated country-specific standards (30), analyzing 12,759 Mexican live births from the WHO Multi-country Survey on Maternal and Newborn Health in 2010 (31). Also, these authors excluded large for gestational age live births from the denominator which would lead to higher SGA rates. Also, missing values from the National Registry would slightly underrepresent SGA outcomes in this study. Lastly, there were improvements in maternal and neonatal public health in Mexico in the seven years following Ota's study (32).

We observed a significant association between SGA and mothers living in settings above 2,000 m. The states with the highest average altitude, namely the State of Mexico (2,350 m), Tlaxcala (2,340 m), and Mexico City (2,247 m) (33) presented higher SGA rates, 9.4, 8.0, and 8.8 than their counterparts. The exception was Yucatán which is mostly at sea level (9 m) and presented a rate of 9.4 probably because of its high Mayan ancestry (34). These results are consistent with Buekens et al. (22) who hypothesized that gravid women living in high altitudes in Mexico had lower placental circulation and a higher risk of low birth weight. The effect of high altitude on SGA has been reported in previous studies independently of other risk factors such as economic status (35, 36), but further analysis at the district level is needed to better explain this phenomenon.

Our results show that maternal education, maternal age, marital status, parity, socioeconomic conditions, and antenatal care were associated with SGA. These results provide yet more evidence that social deprivation is associated with poor health outcomes (37). For example, living in poverty is associated with lower levels of education, unplanned pregnancies, stress, and less access to health care facilities. Despite attending school, women living in social deprivation have deficient learning achievement, as well as, social, cultural, and administrative obstacles that reproduce health inequities to their offspring (38). Additionally, we found a significant association between increasing maternal age and SGA in Mexico. This observation is consistent with other studies (39) where advanced maternal age (especially ≥40 years) has been associated with SGA.

We have quantified coverage of and variations in SGA in Mexico. Our analysis points out the need for a new focus on women's health which must address social determinants to reduce SGA. This should include prioritizing the first contact with health care services during the first trimester and the adequate number of antenatal care visits as crucial indicators, providing an opportunity to detect and treat prenatal complications, and for pregnant women to start micronutrient supplements (26). In the long term, further commitment is needed to promote social development, economic policies, and political action to reduce SGA in future generations (40).

## Conclusions

Maternal education, extreme maternal ages, parity, and social deprivation play a significant role in SGA in Mexico. Health care workers should take advantage of this information to personalize preventive services and give tailored counseling for reproductive health throughout the life course. Additionally, Mexican authorities should increase the proportion of live births with a record of weight at birth which could be done by facilitating the continuous training of users to improve the quality of data. The inclusion of critical variables such as maternal body mass index before pregnancy, paternal information, and survival follow-up in national databases would enable more robust analyses in future cohorts. Finally, women's health should be defined beyond the traditional approach, not only addressing medical care attention but also underlying welfare determinants such as education, social inclusion, security, and substantial improvements in data collection.

## Data Availability Statement

The data presented in the study are deposited in the following repository https://github.com/MarioCortinaBorja/Births_in_Mexico_2017.

## Author Contributions

LS contributed with conceptualization, data curation, and writing the original draft. MC supervised data curation, data analysis, and supported writing. HB supported the investigation process, critical review, and editing. EO supported with critical review and editing of the manuscript. All authors contributed to the article and approved the submitted version.

## Funding

LS was supported by a Chevening scholarship MXCV-2019-2522 and conducted this work in partial fulfillment of an MSc in Pediatrics and Child Health at the UCL Institute of Child Health. This research was supported by the National Institute for Health Research (NIHR), Great Ormond Street Hospital Biomedical Research Center. The views expressed are those of the authors and not necessarily those of the National Health Service (NHS), the NIHR, or the UK Department of Health. The funders had no role in study design, data collection, and analysis, decision to publish, or preparation of the manuscript.

## Conflict of Interest

The authors declare that the research was conducted in the absence of any commercial or financial relationships that could be construed as a potential conflict of interest.

## Publisher's Note

All claims expressed in this article are solely those of the authors and do not necessarily represent those of their affiliated organizations, or those of the publisher, the editors and the reviewers. Any product that may be evaluated in this article, or claim that may be made by its manufacturer, is not guaranteed or endorsed by the publisher.
