# Supplementary material for: Maternal Risk Factors for Small-for-Gestational-Age Newborns in Mexico: Analysis of a Nationwide Representative Cohort
Source: Front Public Health. 2021 Dec 23;9:707078. doi: 10.3389/fpubh.2021.707078 (PMC8732993; doi:10.3389/fpubh.2021.707078)
Supplement: Supplementary file 1 [file Table_1.DOCX]

***Supplementary Material***

# SUPPLEMENTARY TABLES AND FIGURES

| **Variable** | **Description** |
| --- | --- |
| Title | National Information Subsystem of Livebirths |
| Author | Ministry of Health, Mexico |
| Subject | Live births certificates |
| Description | Nominal registry of live births occurred in Mexico and their conditions |
| Publisher | Mexican Government |
| Date | 2017 |
| Resource Type | “text” |
| Format | Microsoft Office Access Database |
| Resource identifier | http://dgis.salud.gob.mx/contenidos/basesdedatos/bdc_nacimientos_gobmx.html |
| Language | Spanish |
| Relation | “IsPartOfAnnualRegistryUpdates” |
| Coverage | 2017 |
| Rights Management | “Open Access” |

Table S1. Dublin core description

| **Variables** | **Pregnancies** | **Percentage (%)** |
| --- | --- | --- |
| *n****: 2,064,507*** |  |  |
| Data on number of babies | 2,060,069 | 99.79 |
| Twins | 33,182 | 1.61 |
| three or more | 957 | 0.05 |
| Singletons | 2,025,930 | 98.13 |
| Missing | 4,438 | 0.21 |
| Weight at birth | 1,951,125 | 94.51 |
| <2500 | 125,534 | 6.08 |
| ≥2500 | 1,825,591 | 88.43 |
| Missing | 113,382 | 5.49 |
| Schooling, years | 2,017,842 | 97.74 |
| No studies | 40,954 | 1.98 |
| 1 to 6 | 261,935 | 12.69 |
| 7 to 9 | 775,383 | 37.56 |
| 10 to12 | 597,478 | 28.94 |
| More than 12 | 359,633 | 17.42 |
| Missing | 29,124 | 1.41 |
| Maternal age group, years | 2,063,576 | 99.95 |
| Less than 19 | 282,753 | 13.7 |
| 19 to 34 | 1,576,593 | 76.37 |
| More or equal 35 | 204,230 | 9.89 |
| Missing | 931 | 0.05 |
| Parity | 2,064,075 | 99.98 |
| First pregnancy | 749,842 | 36.32 |
| Second or more pregnancies | 1,314,233 | 63.66 |
| Missing | 432 | 0.02 |
| Marital status | 2,015,663 | 97.63 |
| Single | 207,517 | 10.05 |
| Married | 1,808,146 | 87.58 |
| Missing | 48,844 | 2.37 |
| Social deprivation | 2,059,158 | 99.74 |
| Very low | 289,400 | 14.02 |
| Low | 831,648 | 40.28 |
| Medium | 239,053 | 11.58 |
| High | 356,674 | 17.28 |
| Very high | 342,383 | 16.58 |
| Missing | 5,349 | 0.26 |
| Gestational age | 2,062,422 | 99.9 |
| Preterm | 148,986 | 7.22 |
| Term | 1,913,436 | 92.68 |
| Missing | 2,085 | 0.1 |
| Child sex | 2,062,994 | 99.93 |
| Men | 1,050,800 | 50.9 |
| Women | 1,012,194 | 49.03 |
| Missing | 1,513 | 0.07 |

Table S2. Distribution of baseline characteristics and missing values among all registries reported to SINAC, Mexico 2017.

| **Variables** | **Number of live births by maternal age categories** | | | | | | | |
| --- | --- | --- | --- | --- | --- | --- | --- | --- |
|  | **<19y.o.** | **%** | **19-34 y.o.** | **%** | **35-39** | **%** | **>=40** | **%** |
| Total | 251,928 |  | 1,411,342 |  | 143,504 |  | 34,703 |  |
|  |  |  |  |  |  |  |  |  |
| Schooling, in years |  |  |  |  |  |  |  |  |
| No studies | 4,653 | 1.8 | 24,633 | 1.7 | 4,596 | 3.2 | 1,707 | 4.9 |
| 1 to 6 | 37,954 | 15.1 | 161,997 | 11.5 | 25,479 | 17.8 | 7,676 | 22.1 |
| 7 to 9 | 137,053 | 54.4 | 513,347 | 36.4 | 41,624 | 29.0 | 10,226 | 29.5 |
| 10 to 12 | 70,066 | 27.8 | 440,208 | 31.2 | 27,010 | 18.8 | 5,868 | 16.9 |
| more than 12 | 2,202 | 0.9 | 271,157 | 19.2 | 44,795 | 31.2 | 9,226 | 26.6 |
| Parity |  |  |  |  |  |  |  |  |
| First pregnancy | 207,764 | 82.5 | 438,920 | 31.1 | 17,792 | 12.4 | 3,991 | 11.5 |
| Second or more pregnancies | 44,146 | 17.5 | 972,281 | 68.9 | 125,705 | 87.6 | 30,708 | 88.5 |
| Marital status |  |  |  |  |  |  |  |  |
| Single | 49,401 | 19.6 | 125,607 | 8.9 | 10,944 | 7.6 | 3,356 | 9.7 |
| Married | 202,527 | 80.4 | 1,285,735 | 91.1 | 132,560 | 92.4 | 31,347 | 90.3 |
| Social deprivation |  |  |  |  |  |  |  |  |
| Very low | 32,106 | 12.7 | 199,976 | 14.2 | 24,786 | 17.3 | 6,001 | 17.3 |
| Low | 99,532 | 39.5 | 576,485 | 40.8 | 57,849 | 40.3 | 13,806 | 39.8 |
| Medium | 31,746 | 12.6 | 168,164 | 11.9 | 16,382 | 11.4 | 3,845 | 11.1 |
| High | 44,876 | 17.8 | 242,525 | 17.2 | 22,954 | 16.0 | 5,573 | 16.1 |
| Very high | 43,668 | 17.3 | 224,192 | 15.9 | 21,533 | 15.0 | 5,478 | 15.8 |
| ANC, by trimester |  |  |  |  |  |  |  |  |
| No visits | 8,471 | 3.4 | 25,197 | 1.8 | 2,035 | 1.4 | 724 | 2.1 |
| First | 168,399 | 66.8 | 1,099,621 | 77.9 | 118,424 | 82.5 | 27,337 | 78.8 |
| Second | 60,028 | 23.8 | 232,989 | 16.5 | 18,671 | 13.0 | 5,336 | 15.4 |
| Third | 11,852 | 4.7 | 40,233 | 2.9 | 3,090 | 2.2 | 945 | 2.7 |
| ANC, number of visits |  |  |  |  |  |  |  |  |
| No visits | 8,471 | 3.4 | 25,190 | 1.8 | 2,035 | 1.4 | 724 | 2.1 |
| 1 | 4,836 | 1.9 | 14,562 | 1.0 | 1,143 | 0.8 | 351 | 1.0 |
| 2 | 7,108 | 2.8 | 24,766 | 1.8 | 1,726 | 1.2 | 523 | 1.5 |
| 3 | 13,082 | 5.2 | 45,811 | 3.2 | 3,490 | 2.4 | 998 | 2.9 |
| 4 | 21,363 | 8.5 | 80,490 | 5.7 | 6,222 | 4.3 | 1,707 | 4.9 |
| ≥5 | 192,939 | 76.6 | 1,199,163 | 85.0 | 126,685 | 88.3 | 29,805 | 85.9 |
| Altitude |  |  |  |  |  |  |  |  |
| Low (<80m) | 34,330 | 13.6 | 203,648 | 14.4 | 19,623 | 13.7 | 4,513 | 13.0 |
| Mid (80-1,999m) | 145,491 | 57.8 | 787,296 | 55.8 | 79,862 | 55.7 | 19,227 | 55.4 |
| High (≥2,000m) | 72,107 | 28.6 | 420,398 | 29.8 | 44,019 | 30.7 | 10,963 | 31.6 |
| Centiles |  |  |  |  |  |  |  |  |
| Non-SGA (>10p) | 230,592 | 91.5 | 1,320,862 | 93.6 | 135,122 | 94.2 | 32,294 | 93.1 |
| SGA (<10p) | 21,336 | 8.5 | 90,480 | 6.4 | 8,382 | 5.8 | 2,409 | 6.9 |
| Term/Preterm |  |  |  |  |  |  |  |  |
| Term | 236,542 | 93.9 | 1,330,897 | 94.3 | 132,056 | 92.0 | 31,253 | 90.1 |
| Preterm | 15,386 | 6.1 | 80,445 | 5.7 | 11,448 | 8.0 | 3,450 | 9.9 |

Table S3. Distribution of baseline characteristics of included live births according to fine maternal age categories. Mexico 2017

| **Variables** | **Number of live births by sex of the child** | | | |
| --- | --- | --- | --- | --- |
|  | **Male** | **%** | **Female** | **%** |
| Total | 949,902 |  | 891,575 |  |
|  |  |  |  |  |
| Schooling, in years |  |  |  |  |
| No studies | 18,444 | 1.9 | 17,145 | 1.9 |
| 1 to 6 | 120,958 | 12.7 | 112,148 | 12.6 |
| 7 to 9 | 362,120 | 38.1 | 340,130 | 38.1 |
| 10 to 12 | 280,198 | 29.5 | 262,954 | 29.5 |
| more than 12 | 168,182 | 17.7 | 159,198 | 17.9 |
| Parity |  |  |  |  |
| First pregnancy | 347,545 | 36.6 | 320,922 | 36.0 |
| Second or more pregnancies | 602,275 | 63.4 | 570,565 | 64.0 |
| Marital status |  |  |  |  |
| Single | 98,402 | 10.4 | 90,906 | 10.2 |
| Married | 851,500 | 89.6 | 800,669 | 89.8 |
| Social deprivation |  |  |  |  |
| Very low | 135,099 | 14.2 | 127,770 | 14.3 |
| Low | 384,958 | 40.5 | 362,714 | 40.7 |
| Medium | 113,350 | 11.9 | 106,787 | 12.0 |
| High | 163,709 | 17.2 | 152,219 | 17.1 |
| Very high | 152,786 | 16.1 | 142,085 | 15.9 |
| ANC, by trimester |  |  |  |  |
| No visits | 19,041 | 2.0 | 17,386 | 2.0 |
| First | 727,914 | 76.6 | 685,867 | 76.9 |
| Second | 164,627 | 17.3 | 152,397 | 17.1 |
| Third | 28,973 | 3.1 | 27,147 | 3.0 |
| ANC, number of visits |  |  |  |  |
| No visits | 19,037 | 2.0 | 17,383 | 1.9 |
| 1 | 10,853 | 1.1 | 10,039 | 1.1 |
| 2 | 17,872 | 1.9 | 16,251 | 1.8 |
| 3 | 32,867 | 3.5 | 30,514 | 3.4 |
| 4 | 57,204 | 6.0 | 52,578 | 5.9 |
| ≥5 | 797,412 | 83.9 | 751,180 | 84.3 |
| Altitude |  |  |  |  |
| Low (<80m) | 134,270 | 14.1 | 127,844 | 14.3 |
| Mid (80-1,999m) | 531,310 | 55.9 | 500,566 | 56.1 |
| High (≥2,000m) | 284,322 | 29.9 | 263,165 | 29.5 |
| Centiles |  |  |  |  |
| Non-SGA (>10p) | 870,982 | 91.7 | 847,888 | 95.1 |
| SGA (<10p) | 78,920 | 8.3 | 43,687 | 4.9 |
| Term/Preterm |  |  |  |  |
| Term | 889,810 | 93.7 | 840,938 | 94.3 |
| Preterm | 60,092 | 6.3 | 50,637.0 | 5.7 |

Table S4. Distribution of baseline characteristics of included live births according to the sex of the child. Mexico 2017

| **Raking** | **State** | **Abbrev** | **Non-SGA** | **%** | **SGA** | **%** | **Term-SGA** | **%** | **Preterm-SGA** | **%** | **Total** |
| --- | --- | --- | --- | --- | --- | --- | --- | --- | --- | --- | --- |
| 1 | State of Mexico | Mex | 221,979 | 90.6 | 23,059 | 9.4 | 21,387 | 8.7 | 1,672 | 0.7 | 245,038 |
| 2 | Yucatán | Yuc | 27,027 | 90.6 | 2,796 | 9.4 | 2,514 | 8.4 | 282 | 0.9 | 29,823 |
| 3 | Guerrero | Gro | 47,133 | 91.2 | 4,552 | 8.8 | 4,284 | 8.3 | 268 | 0.5 | 51,685 |
| 4 | Mexico City | DF | 92,489 | 91.2 | 8,876 | 8.8 | 8,130 | 8.0 | 746 | 0.7 | 101,365 |
| 5 | Puebla | Pue | 101,133 | 91.7 | 9,120 | 8.3 | 8,511 | 7.7 | 609 | 0.6 | 110,253 |
| 6 | Chiapas | Chis | 72,687 | 91.8 | 6,460 | 8.2 | 6,131 | 7.7 | 329 | 0.4 | 79,147 |
| 7 | Tlaxcala | Tlax | 20,805 | 92.0 | 1,821 | 8.0 | 1,703 | 7.5 | 118 | 0.5 | 22,626 |
| 8 | Oaxaca | Oax | 58,157 | 92.4 | 4,814 | 7.6 | 4,542 | 7.2 | 272 | 0.4 | 62,971 |
| 9 | Hidalgo | Hgo | 38,036 | 92.7 | 3,010 | 7.3 | 2,815 | 6.9 | 195 | 0.5 | 41,046 |
| 10 | Morelos | Mor | 25,555 | 92.7 | 2,020 | 7.3 | 1,854 | 6.7 | 166 | 0.6 | 27,575 |
| 11 | Queretaro | Qro | 34,207 | 93.2 | 2,512 | 6.8 | 2,291 | 6.2 | 221 | 0.6 | 36,719 |
| 12 | Quintana Roo | QR | 23,114 | 93.3 | 1,656 | 6.7 | 1,534 | 6.2 | 122 | 0.5 | 24,770 |
| 13 | Michoacán | Mich | 74,212 | 93.5 | 5,145 | 6.5 | 4,774 | 6.0 | 371 | 0.5 | 79,357 |
| 14 | Campeche | Camp | 11,970 | 93.6 | 813 | 6.4 | 750 | 5.9 | 63 | 0.5 | 12,783 |
| 15 | Zacatecas | Zac | 25,498 | 93.9 | 1,661 | 6.1 | 1,461 | 5.4 | 200 | 0.7 | 27,159 |
| 16 | Veracruz | Ver | 94,986 | 94.0 | 6,082 | 6.0 | 5,692 | 5.6 | 390 | 0.4 | 101,068 |
| 17 | Guanajuato | Gto | 101,283 | 94.1 | 6,304 | 5.9 | 5,687 | 5.3 | 617 | 0.6 | 107,587 |
| 18 | Tabasco | Tab | 38,070 | 94.2 | 2,362 | 5.8 | 2,133 | 5.3 | 229 | 0.6 | 40,432 |
| 19 | Aguascalientes | Ags | 23,498 | 94.2 | 1,439 | 5.8 | 1,288 | 5.2 | 151 | 0.6 | 24,937 |
| 20 | San Luis Potosí | SLP | 40,280 | 94.4 | 2,386 | 5.6 | 2,157 | 5.1 | 229 | 0.5 | 42,666 |
| 21 | Jalisco | Jal | 120,649 | 94.4 | 7,146 | 5.6 | 6,567 | 5.1 | 579 | 0.5 | 127,795 |
| 22 | Durango | Dgo | 29,979 | 95.0 | 1,567 | 5.0 | 1,431 | 4.5 | 136 | 0.4 | 31,546 |
| 23 | Tamaulipas | Tamps | 47,826 | 95.1 | 2,457 | 4.9 | 2,223 | 4.4 | 234 | 0.5 | 50,283 |
| 24 | Coahuila | Coah | 48,610 | 95.2 | 2,451 | 4.8 | 2,207 | 4.3 | 244 | 0.5 | 51,061 |
| 25 | Nayarit | Nay | 17,141 | 95.5 | 805 | 4.5 | 736 | 4.1 | 69 | 0.4 | 17,946 |
| 26 | Chihuahua | Chih | 52,556 | 95.7 | 2,359 | 4.3 | 2,141 | 3.9 | 218 | 0.4 | 54,915 |
| 27 | Colima | Col | 9,779 | 95.7 | 436 | 4.3 | 407 | 4.0 | 29 | 0.3 | 10,215 |
| 28 | Baja California Sur | BCS | 9,525 | 95.8 | 416 | 4.2 | 384 | 3.9 | 32 | 0.3 | 9,941 |
| 29 | Nuevo León | NL | 82,055 | 96.0 | 3,451 | 4.0 | 3,076 | 3.6 | 375 | 0.4 | 85,506 |
| 30 | Baja California | BC | 47,466 | 96.1 | 1,946 | 3.9 | 1,761 | 3.6 | 185 | 0.4 | 49,412 |
| 31 | Sinaloa | Sin | 43,205 | 96.7 | 1,465 | 3.3 | 1,341 | 3.0 | 124 | 0.3 | 44,670 |
| 32 | Sonora | Son | 37,960 | 96.9 | 1,220 | 3.1 | 1,095 | 2.8 | 125 | 0.3 | 39,180 |
| NATIONAL | | | 1,718,870 | 93.3 | 122,607 | 6.7 | 113,007 | 6.1 | 9,600 | 0.5 | 1,841,477 |

Table S5. Proportions of SGA, Term-SGA and Preterm-SGA live births in Mexico, according to the State of mother´s residence. Mexico, 2017

| **Gestational age, category in completed weeks** | **Non-SGA** | **%** | **SGA** | **%** |
| --- | --- | --- | --- | --- |
| <28 | 3,070 | 0.2 | 122 | 0.1 |
| 28-31 | 7,051 | 0.4 | 773 | 0.6 |
| 32-36 | 91,008 | 5.3 | 8,705 | 7.1 |
| 37-41 | 1,604,612 | 93.4 | 108,611 | 88.6 |
| 42 | 13,129 | 0.8 | 4,396 | 3.6 |
| Total | 1,718,870 | 100.0 | 122,607 | 100.0 |

Table S6. Proportions of SGA, and Non-SGA according to gestational age categories. Mexico, 2017
